# Participatory Surveillance and Candidacy: A Discourse Analysis of Views on Self-Testing for Proteinuria in Pregnancy

**DOI:** 10.1177/10497323241274270

**Published:** 2024-10-17

**Authors:** Bethany E. Jakubowski, Katherine L. Tucker, Layla Lavallee, Hannah Wilson, Lucy Mackillop, Lucy C. Chappell, Richard J. McManus, Lisa Hinton

**Affiliations:** 1 Department of Women and Children’s Health, 4616 King’s College London, London, UK; 2 Department of Health Services Research and Policy, London School of Hygiene and Tropical Medicine, Oxford, UK; 3 Nuffield Department of Primary Care Health Sciences, 6396 University of Oxford, Oxford, UK; 4 Nuffield Department of Women’s and Reproductive Health, 6396 University of Oxford, Oxford, UK

**Keywords:** antenatal care;, candidacy;, empowerment;, self-testing;, self-management;, hypertension

## Abstract

Actively involving people in self-monitoring and management during their pregnancy is an emerging clinical and social practice. Self-monitoring of blood pressure and self-testing for proteinuria, key diagnostic tests for pre-eclampsia, are becoming commonplace in hypertensive pregnancies. While evidence exists on the acceptability and feasibility of self-monitoring blood pressure, evidence for self-testing for proteinuria in pregnancy is thin, with little knowledge of how it might affect the traditional structures of maternity care. As part of a diagnostic accuracy study on self-testing for proteinuria, pregnant people and healthcare professionals were recruited to a qualitative study to understand their experiences of, and attitudes to, self-testing. Multiple qualitative methods were used, including interviews, focus groups, and free text postcards. A discourse analysis was conducted to understand how self-testing might inform and reshape routine antenatal care. Analysis revealed a tension between the empowering concept of participatory surveillance, which pregnant people and healthcare professionals were broadly positive about, and the adjudications made by healthcare professionals about the candidacy, or *suitability*, of certain pregnant people to self-test. Candidacy is a framework for understanding what influences access to healthcare for socially disadvantaged groups, including professional judgments that impact access to interventions. While participatory surveillance was felt to have the potential to empower pregnant people in antenatal care, the loss of the traditional clinical gaze was disquieting for some, and pregnant people and healthcare professionals were reluctant to cede professional responsibility.

## Introduction

### Background

The primary aim of the antenatal care pathway in high-income countries is to ensure safe and positive outcomes for the parent and baby. While many pregnancies are uncomplicated, and a natural, normal phase in a person’s life, complications can develop quickly and require either continuous surveillance or medical intervention. Antenatal care provides vital surveillance to monitor for, and respond quickly to, those changes. For uncomplicated pregnancies, the World Health Organization (WHO) recommends a minimum of eight antenatal appointments, the purpose of which is to reduce perinatal mortality and improve pregnant people’s experience of care (WHO, 2016). In the United Kingdom, the National Institute for Health and Care Excellence (NICE) recommends ten for nulliparous pregnant people and eight for parous pregnant people (National Institute for Health and Care Excellence [NICE], 2008). More appointments may be necessary if the pregnant person experiences complications, such as gestational diabetes, and a greater level of surveillance is required. As such, surveillance during pregnancy, particularly for high-risk pregnancies, is widely normalized. Pregnant people, motivated by a desire to protect their unborn baby, are expected to engage in risk-averse behavior and self-surveillance, often at the request of their healthcare professionals (Jakubowski, Hinton, et al., 2022; D. A. Lupton, 2011; D. Lupton, 2012).

Hypertensive disorders affect around 10% of pregnancies worldwide (Say et al., 2014; Wang et al., 2021) and 8%–10% of pregnancies in the United Kingdom (National Institute for Health and Care Excellence [NICE], 2019). Pre-eclampsia, a serious hypertensive disorder that causes significant maternal morbidity and mortality globally (Say et al., 2014), is characterized by hypertension (high blood pressure) and multi-organ features including proteinuria (the leaking of protein into urine) and other maternal and fetal complications (Brown et al., 2018). Blood pressure and the presence of dipstick proteinuria are routinely monitored by healthcare professionals at antenatal appointments. Self-testing of proteinuria, when combined with self-monitoring blood pressure, has the potential to improve the screening for pre-eclampsia and provide pregnant people with more autonomy and involvement in their care. While there is an emerging body of both quantitative and qualitative evidence regarding the safety, efficacy, and acceptability of home blood pressure monitoring (Chappell et al., 2022; Hinton et al., 2017; Kitt et al., 2023; Pealing et al., 2022; Tucker et al., 2022), the evidence for self-testing for proteinuria is thin (Tucker et al., 2018). Prior to the COVID-19 pandemic, the evidence base for home blood pressure monitoring was still being developed, and while some pregnant people had adopted home blood pressure monitoring (Hinton et al., 2017; Paterson et al., 2023; Tucker et al., 2021), self-testing of urine remained largely unexplored. The pandemic accelerated the need for remote care; self-monitoring of blood pressure was rapidly implemented by some hospitals and trusts (Wilson et al., 2022), and a considerable proportion of antenatal care appointments switched to virtual or telephone consultations (Hinton et al., 2024; Jardine et al., 2021). Yet, there remains a pressing need for evidence on remote monitoring in post-pandemic care pathways, where face-to-face care has largely resumed.

#### Candidacy

Candidacy describes how eligibility for medical services is jointly negotiated between healthcare seekers and health services (Dixon-Woods et al., 2006) and was initially developed to synthesize our understanding of how socially disadvantaged groups access healthcare. This theory is particularly apposite to maternity care where widening inequalities are responsible for increasingly poorer outcomes in marginalized groups (Knight et al., 2022). Candidacy, in relation to self-management in pregnancy, has not been extensively researched, but recent studies have used candidacy as a lens to explore other aspects of maternity care. Remote care can reshape how candidacy is negotiated in maternity care and risks exacerbating already prevalent inequalities (Hinton et al., 2023). As Hinton and colleagues argue, some people find remote consultations difficult to access, for example, if they do not have sufficient digital access or health literacy. Those who have very little social capital have fewer tools to negotiate remote care, are less able to engage in its new responsibilities, and thus risk further disadvantage (Hinton et al., 2023). Pregnant people who are denied choice in their antenatal care are more likely to experience paternalistic care and discrimination and view antenatal care as a system of surveillance, rather than support, which subsequently impacts their engagement and candidacy (Rayment-Jones et al., 2019).

#### Participatory Surveillance

Foucault described surveillance as a system of constant registration and inspection, a combination of continual gaze and monitoring, or a regulation of actions (Goodyear et al., 2019). Traditional models of surveillance, like Foucault’s, describe top-down, hierarchical approaches that emphasize surveillance as “them upon us” (Vaz & Bruno, 2003); the object of the surveillance is powerless and/or passive. In contrast, in participatory surveillance, the object is both willing and potentially empowered by the surveillance (Albrechtslund, 2008). The clinical monitoring of high-risk pregnancies has echoes of traditional surveillance (Hinton et al., 2021, where anxieties over the safety of the pregnant person and the baby underpin a system of clinical monitoring that pregnant people are expected to engage with to ensure the health and safety of their baby (Coxon et al., 2012; Lupton, 2012). Participatory surveillance is a concept with roots in digital and public health, and has rarely been applied to antenatal care research, but in practice, it is becoming more commonplace as traditional surveillance activities carried out by healthcare professionals, such as blood pressure monitoring and urine testing, are slowly being shared with pregnant people (Fletcher et al., 2021; Wilson et al., 2022).

This paper explores healthcare professionals and pregnant people’s views on self-testing, whether it should be integrated into antenatal care, and how antenatal care surveillance, traditionally carried out by healthcare professionals, can be shared with pregnant people.

## Methods

### Data Collection

This qualitative study took place alongside a diagnostic accuracy study of self-testing urine in pregnancy (the Urine Detection in Pregnancy study—UDIP, *n* = 335 participants) (Jakubowski, Stevens, et al., 2022), which found that pregnant people can dipstick test and read proteinuria results from their urine as accurately as both healthcare professionals and an automated dipstick reader, compared to a laboratory standard (Jakubowski, Stevens, et al., 2022). The UDIP study was conducted in National Health Service (NHS) settings in the United Kingdom. Semi-structured (in person or telephone) one-on-one interviews with pregnant people and healthcare professionals and focus groups with healthcare professionals were conducted alongside the diagnostic accuracy study. All interviews and focus groups were facilitated by the first author, BJ. Additionally, all pregnant people in the UDIP study were provided a free text response postcard in their study packs and were invited to share their thoughts on the self-testing process once they had completed it. Free text postcards support access to persons who are not normally heard in research (Thorpe & Holt, 2008), and in this study, postcards were included to ensure a wider range of participants’ views were captured over the course of the study.

#### Inclusion Criteria

Pregnant people were eligible for the study if they had taken part in the UDIP study, which required them to have a finding of raised blood pressure or diagnosis of hypertension, were ≥20 weeks’ gestation, and were ≥18 years of age. Healthcare professionals were eligible if they were part of the UDIP participant’s usual care team, had cared for pregnant persons who had participated in the study, had experience of managing pregnant people who self-monitor blood pressure during pregnancy, or were working in an NHS trust. Pregnant people were purposively sampled and were approached by a research midwife, they were provided with study information, and, if willing to take part, signed a consent form ahead of the interview. Healthcare professionals were also approached with the support of research midwives and recruited through a mixture of purposive and snowball sampling.

#### Topic Guides

An iterative approach was used to develop a topic guide. Questions and prompts were originally developed with the study team from a review of existing literature and re-defined over the course of the study to explore some of the responses and concerns raised by the participants in more depth.

#### Ethical Approval

The UDIP study was approved by the Yorkshire and the Humber – Leeds East Research Ethics Committee (Reference: 18/YH/0208). All participants provided written informed consent prior to enrollment in the study.

### Data Analysis

The interviews and focus groups were audio recorded and, alongside the free text postcards, were transcribed verbatim by an independent transcriber and proofread for accuracy. Analysis began with a detailed examination of the transcripts, identifying initial themes and developing an understanding of the material. The transcripts were then uploaded to NVivo 12 Pro (QSR International Pty Ltd, released 2018). An initial round of coding was conducted using Braun and Clarke’s guidelines (Braun & Clarke, 2006) for thematic analysis. After the first round of coding, these inductive themes were mapped using the “one sheet of paper” (OSOP) method (Ziebland & McPherson, 2006) to visualize the data as a whole. This visualization suggested that thematic analysis and the OSOP method did not support sufficient exploration of the discourses that were present in the data. At this stage, there was evidence of complex and contradictory discourses in the data regarding empowerment, candidacy, and surveillance, and the study team decided that to fully explore the complexities of the discourses, a discourse analytic approach would be more appropriate.

#### Discourse Analysis

Discourse analysis involves closely studying language to understand *how* topics are spoken about (Carabine, 2001). Discourse analysis enables us to understand how language is being used within larger social and cultural contexts and to study power, resistance, contests, and struggles (Wetherell et al., 2001). We used Carabine’s Foucauldian approach to discourse analysis; this type of discourse analysis explores the mechanisms of power, and the relationship between power and knowledge. While a Foucauldian approach is useful in understanding how power, knowledge, and surveillance co-exist and interact, Foucault’s theories of power and surveillance were not primarily used in this discourse analysis. The purpose of this analysis was to explore the *exchange* of knowledge and power between healthcare professionals and patients, facilitated by self-testing. This analysis afforded the space to understand how the sharing of medical knowledge, customarily held by healthcare professionals, disrupts traditional models of power in healthcare systems and results in a more participatory form of surveillance.

Shaw and Bailey situate discourse analysis on three levels: micro, meso, and macro (Shaw & Bailey, 2009). The meso level approach they describe, where this study was situated, focuses on the connections between language and wider social contexts. Situating this study at the meso level allowed for an exploration of how self-testing challenges existing power structures in antenatal care and how a concept such as participatory surveillance can exist within a system where surveillance is traditionally performed by healthcare professionals.

Research questions that arose after the initial thematic and OSOP analysis included: how does self-testing challenge traditional power structures in antenatal care? How do paternalism and medicalization interact with participatory surveillance in these data? How is candidacy being negotiated by healthcare professionals and pregnant people in these data? How and why is participatory surveillance being characterized as empowering? These questions proceeded to guide the discourse analysis.

The candidacy theory, specifically the concept of adjudications, was identified as a discourse in these data. Discourse analysis created space for the consideration of language, which was particularly pertinent to candidacy, as *how* healthcare professionals talked about their patients contributed to the authors understanding of paternalism in these data. The candidacy discourse created a framework in which to explore the second discourse identified in these data—participatory surveillance. As candidacy is a theory which helps to understand how people engage with, access, and navigate care, exploring this discourse created space for a deeper exploration of how eligibility for participatory surveillance, in this case self-testing, is negotiated between healthcare professionals and pregnant people. Discourse analysis also allowed the authors to understand and explore the contradictions in these data between paternalism and empowerment, which was reflected in the candidacy discourse, and the conceptualization of surveillance, which was reflected in the participatory surveillance discourse.

The three methods of data collection, interviews, focus groups, and postcards, were initially coded together to understand the breadth of the dataset. As the analysis developed, the postcards were considered in isolation to ensure the discourses were reflected across all datasets. As expected, the interviews and focus groups provided more depth and nuance; therefore, the coding framework was mapped solely to the postcard data to ensure that, while the data were shallow, the discourses that were strongly present in the interviews and focus groups were also present in the postcards. The coding was led by BJ, but it was a collaborative process; the framework was regularly presented to the study team at trial meetings (LH, KT, RM, and LC). LH closely oversaw the coding process and regularly met with BJ to refine themes and, later, discourses.

## Results

A total of 21 pregnant people agreed to be interviewed. Of the 21 interviews, 19 were conducted in person and two by telephone. Most of the in-person interviews were conducted in NHS hospitals either at the bedside, as some participants were inpatients, or after scheduled antenatal appointments. Two took place in participants’ homes as they were postnatal. The length of the interviews ranged from 5 min to 1 hr, often reflective of the interview setting (those that took place in participants’ homes were longer). These data were primarily collected in late 2019 and early 2020, prior to the COVID-19 pandemic. The mean age of interview participants was 36 years, the mean gestational age at the time of recruitment to the (UDIP) study was 31 weeks (participants’ gestational age at interview was not recorded), and the majority were nulliparous. 52% of participants were white British, 10% of participants were Asian or Asian British, and 10% were Black or Black British, with the rest drawn from other ethnicities (Table 1). A total of 106 free text postcards were collected from pregnant people during the study across the three study sites.

**Table 1.** Demographics of pregnant participants (interviews *n* = 21).

	Median	IQR
Age	36	32–40
Gestational age at recruitment in weeks	33	27–36
	Frequency	%
First pregnancy	12	57.1
Ethnicity		
Asian or Asian British	3	14.3
Black or Black British	2	9.6
Mixed	1	5.8
White British	11	52.4
Other	4	19.0

Eighteen healthcare professionals agreed to be interviewed (Table 2), and all interviews were conducted in person. Five focus groups with healthcare professionals were conducted with 15 participants (Table 2). A mix of healthcare professionals were recruited: midwives, community midwives, obstetricians, junior doctors, and healthcare assistants. The length of interviews ranged from 5 min to half an hour, and the length of focus groups ranged from 10 to 25 min.

**Table 2.** Summary of Interview and Focus Group Participants (Healthcare Professionals).

Professional Role	Number of Participants (Interview)	Number of Participants (Focus Group)
Midwives	10	7
Junior doctors	6	3
Consultants	1	3
Allied healthcare professionals	1	1
Medical students	0	1

Self-testing was found to be broadly acceptable to healthcare professionals and pregnant people. While these results indicated there is space for self-testing in antenatal care, the analyses shed light on the complexities of negotiating empowerment and candidacy. Two discourses were identified in the data and are used here to explore the concept of self-testing—candidacy and participatory surveillance.

### Empowerment in Tension: Participatory Surveillance and Patient Autonomy

Most of the pregnant people in this dataset were considered to have higher risk pregnancies, having been diagnosed with gestational hypertension or pre-existing chronic hypertension. Self-testing for these individuals was broadly acceptable to both them and their healthcare professionals.

I think at home [testing] would potentially be quite useful especially for out in the community if women could (yeah) test their urine at home and then come to their midwife appointment saying whatever the results is. (Healthcare Professional (HCP) Interview 5, midwife)

Yes, I think I would be, (yeah) pretty much fine because I could record the result and then just pass it onto, onto them with the next appointment or phone them and let them know the results. Yeah, I would be, I think I would be ninety-nine per cent confident how to do it in, in the future if I had to do it myself. (Pregnant Participant (PP) Interview 5)

Traditional surveillance in pregnancy can be characterized by the Foucauldian concept of “them upon us,” but a new trend has been emerging in recent years, accelerated by the COVID-19 pandemic (Paterson et al., 2023; Wilson et al., 2022), that potentially shifts the burden of surveillance activities to pregnant people, creating a more participatory form of surveillance. In these data, the acceptability of self-testing among both pregnant people and clinicians illustrates the extent the narrative has changed. Participatory surveillance involves a willing and engaged person who is empowered by the surveillance; in these data, one of the benefits of self-testing for pregnant people was a heightened sense of involvement and control during their pregnancy.

Albrechtslund theorized that participatory surveillance and empowerment are intertwined (Albrechtslund, 2008), and this is highlighted in the following excerpt from an interview with a pregnant participant, who was an inpatient at the time.

Respondent: No, I mean, I didn’t have a lot of midwife appointments anyway and my midwife ended up going off long term sick, so (um) that wasn’t really an avenue that was open to very much because she wasn’t available. (um) But I was seeing consultants and stuff because I was already considered high-risk and I would still have gone to those appointments, obviously. (um) I might have felt like I was a bit more of a participant in my pregnancy rather than a patient. (laughs)

Interviewer: Sure.

Respondent: Because you do, especially being in hospital, you don’t have any independence.

Interviewer: Yeah.

Respondent: Everything’s kind of done for you but (um) and having some sort of autonomy early on would just be nice for your mental health because you sort of feel like you’re part of looking after your health. (PP Interview 7)

Notably, this individual wanted to feel like more of a “participant” in their pregnancy rather than a “patient.” There are challenges in the pregnant population regarding the use of the term patient and the medicalization it invokes. This creates linguistic problems. The perspective of the pregnant person is important to consider; do they consider themselves to be a patient and does that differ based on how medicalized their pregnancy has become? This participant has highlighted that tension; they would prefer not to be a “patient,” despite the fact their pregnancy is categorized as “high risk,” and instead, they desire a more active, participatory role in their care, to “look[*ing*] after [their] health.” Most participants in this study were experiencing high risk, and therefore more medicalized, pregnancies which impacted their perception of autonomy and eligibility for an intervention such as self-testing.

Participatory surveillance through self-testing was also considered to be clinically beneficial. Hypertension can emerge very rapidly during pregnancy and risks the health of the pregnant person and the baby if it is severe or progresses to pre-eclampsia. Self-testing for proteinuria provides additional surveillance for pre-eclampsia, which was reassuring to this pregnant person, particularly alongside other self-monitoring activities such as blood pressure monitoring.

Not just when you have an appointment because, I mean some people start developing hypertension, you know, during pregnancy without knowing that they are developing it, so it’s good to sort of be on top of it. (PP Interview 14)

The following extract underscores a healthcare professional’s concerns about the rapid onset of pre-eclampsia and the possibility that self-testing could give pregnant people an incentive to check in with their antenatal care team and create an additional safety net.

So being able to just have that second [inaudible] even if you’re only doing it every couple of days or however it, it’s recommended, it just gives you that extra kind of line of defence because there are no symptoms to pre-eclampsia. It can be absolutely fine, oh seizure. So, something else that [pause] and people don’t call their midwives; they don’t call the hospital because they don’t want to bother anybody. (PP Interview 7)

However, there is also evidence in these data that pregnant people were reluctant to forgo the more traditional form of the clinical gaze (which they often found reassuring)—healthcare professionals carrying out the surveillance activities.

Yeah, I wouldn’t mind. I mean, if it was a thing of cross references, so I test at home and then I don’t know, brought the results in or, and then you guys test it in the hospital and then match them up just to make sure that, you know, for myself or whoever it is doing it at home, they’re doing it accurately or as close as possible. (PP Interview 11)

Empowerment itself is in tension here; both healthcare professionals and pregnant people see the value of empowering pregnant persons and see self-testing as a step towards that concept. But neither is willing to lose or relinquish the traditional clinical gaze. The loss of clinical oversight made some pregnant participants anxious, and the responsibility of self-testing also contributed to their anxiety. Self-testing was seen by some in this study as an amplification of the clinical gaze, even though inviting people to self-test shifts the responsibility for that surveillance to them. For some, this was worrisome; they articulated a tension between the desire for additional surveillance and concerns about who held ultimate responsibility for it. For healthcare professionals, self-testing represents a fundamental shift in how healthcare is practiced, and these data indicate there is hesitancy around the implications of sharing surveillance activities with pregnant people. This was a strong deterrent for one pregnant person, who said seeing the positive proteinuria result made them anxious and that the fear of seeing another positive result would stop them from considering home self-testing altogether.

I kind of panicked, said, “Oh my God, if I was home, had to do this, I would panic and that would probably stop me doing it.” (PP Interview 15)

There is tension in these discourses; participatory surveillance has the potential to create a more autonomous space for pregnant people in antenatal care, yet the loss of the traditional clinical gaze was disquieting for some. But self-testing can also be characterized as an extension of the medical gaze, one that is an amplification of traditional surveillance. These data seem to reflect that self-testing is both disruptive and an intensification of antenatal care surveillance. Self-testing was widely acceptable to both groups, but there was tension in these data between the prospect of citizen empowerment and the clinical gaze as it presently exists.

### Candidacy in Tension: Medicalization and Paternalism in Antenatal Care

In these data, there was a noticeable tension between pregnant people and healthcare professionals regarding the negotiation of candidacy for self-testing. Both groups agreed that self-testing was simple and easy to do, although both shared concerns about the interpretation of results, illustrated in the following example collected from free text postcards from participants. Yet, both groups felt that this could be easily overcome with adequate training and support.

I think it would be feasible—many women are used to doing tests at home (e.g., pregnancy and ovulation tests) so I can’t see that this would be very hard for women to do. Only comment is that the “NEG” and “Tr + sp” colours are very similar, so some many find it hard to distinguish between the two. (Postcard: pregnant participant)

A key domain in the candidacy theory is adjudications, which refers to the judgments made by healthcare professionals regarding a patient’s eligibility to access an intervention (Dixon-Woods et al., 2006). Often, those most socially disadvantaged are at risk of being judged “less eligible” for those interventions (Dixon-Woods et al., 2006). This domain is particularly evident in these data as, despite assertions from both groups that self-testing was simple and easy to do, healthcare professionals described the *unsuitable patient* who was considered too “chaotic” to self-test throughout their pregnancy. This judgment encompassed a wide range of personal, social, or economic circumstances; people who were busy, had multiple other children, could not take time off work or did not turn up to their regular appointments. These people were also not considered to be as “capable” of self-testing, and healthcare professionals often contradicted their earlier statements about self-testing being easy and simple to do at home. Healthcare professionals’ views of who should self-test were at odds with those of pregnant people, suggesting that there are different perceptions of suitability for this intervention.

People with chaotic lifestyles who aren’t going to engage with it and just, you know, you see, “She’s very sensible, she’ll, she’ll be able to manage this study,” and then you see people who clearly you just they can’t barely make their regular routine appointments, how they going to deal with the additional like yeah, so I think that. (FG1 (Focus Group 1) P1 (Participant 1), senior midwife)

In several focus groups and interviews, healthcare professionals also considered non-English speakers to be unsuitable for self-testing.

I work with caseload women, so that’s vulnerable women safeguarding, women who don’t, who don’t speak English and my initial thought is, this might work for some but not for all. So, I think that would be my first reservation. (FG2 P1, midwife)

The language in many of these excerpts was othering, suggesting there are certain groups and persons that are considered by healthcare professionals to be unsuitable candidates, or too high risk for self-testing, due to personal, social, or economic circumstances. These adjudications suggest that eligibility for self-testing is complicated by clinicians’ gatekeeping and limiting access to self-testing based on judgments about suitability. There was dissonance in these data; participatory surveillance is intended to be empowering, yet antenatal care can often feel disempowering and overly medicalized to some pregnant people. Pregnant people felt they have very little control during their pregnancy—“everything’s … done for you” (Pregnant Participant Interview 7, quoted above)—and that reclaiming an element of care, such as self-testing, could redress the balance of responsibility.

One participant encapsulated this tension, asserting their candidacy for self-testing, and, by doing so, emphasized how the lack of control over their care made them uncomfortable:

So if I could go home and test it myself and like, self-medicate as such, it takes all the worry out of them and then also I know it’s in my hands and only my hands which I’m quite a control freak and I would prefer that. I really would because especially at the moment, it’s my baby’s life that’s in my hands and I don’t like, the thought of it being in somebody else’s. (PP Interview 4)

This participant was an inpatient, and feeling out of control was a recurring theme in the interview, an encapsulation of the medicalization experienced, particularly by those who are categorized as high risk during their pregnancy. Asserting candidacy for self-testing was complicated; for instance, healthcare professionals were concerned that fears over hospital admission would lead to pregnant people underestimating their results.

(um) I think a lot of people are afraid of what their results might be and if they, if something could cause them to end up as an inpatient in hospital, that they might be a little bit dubious to look at it slightly differently and say, “Oh no, that’s, it’s not that because if it was one over, I know I might end up admitted.” So, you wanted [don’t] want to put their care at risk, if that makes sense? (FG1 P5, midwife)

Healthcare professionals were initially positive about self-testing and the empowerment it could provide pregnant people but would often make judgments during the course of the focus group or interview; some of these adjudications were about clinical suitability, but there was no consensus in these data whether high-risk or low-risk pregnant persons were best suited to self-testing. More often, the adjudications focused on social and cultural attributes—healthcare professionals were making judgments based on personal characteristics, not clinical suitability. In these data, control over an individual’s candidacy illustrates how medicalization in antenatal care can lead to paternalistic, and even discriminatory, behaviors.

I think it’s a good idea but you have to pick the right clients who you think are responsible and capable and will do it accurately and important to keep a link with them, make sure they feedback to you and you act accordingly, yeah. (HCP Interview 14, junior doctor)

Self-testing would represent a shift away from how antenatal care is traditionally practiced, and this was unsettling for some healthcare professionals. Placing the pregnant person at the center of care would disrupt pathways that have been clinically overseen until now, and the strong desire for pregnant people to have more autonomy was complicated by these negotiations of candidacy. There is an inherent contradiction in the desire expressed by healthcare professionals to give control to pregnant people and the reluctance to share the responsibility of self-testing with *all* pregnant people, regardless of personal and social characteristics.

## Discussion

### Main Findings

This paper reports a study of the attitudes of healthcare professionals and pregnant people toward proteinuria self-testing and their thoughts about whether it could be successfully integrated into antenatal care. The two discourses identified in these data, candidacy and participatory surveillance, were in tension. Participatory surveillance reflected the potential for self-testing to be empowering and clinically beneficial, yet both pregnant persons and healthcare professionals were reluctant to surrender the traditional clinical gaze and cede responsibility for surveillance activities to pregnant people. Candidacy as a discourse was evident in judgments about the *unsuitable patient* referred to in these data. The restriction of candidacy resulted in paternalistic, even discriminatory, behaviors, where healthcare professionals were making adjudications of candidacy based on pre-determined social and personal attributes despite a lack of evidence to support these preconceptions. The preconceptions around capability also contrasted with data from the UDIP study which found that pregnant people could self-test as accurately as healthcare professionals and an automated reader (Jakubowski, Stevens, et al., 2022).

### Other Literature

The theory of candidacy helps to frame the paternalistic dynamic described in this dataset between healthcare professionals and pregnant persons. In particular, the construct of adjudications in the theory is a useful lens for these data. This construct suggests that adjudications (judgments) made by healthcare professionals can prevent socially disadvantaged persons accessing, or progressing in, the healthcare system (Dixon-Woods et al., 2006). Those considered unsuitable, or ineligible, for self-testing encompassed a wide group, including anyone who experienced language barriers in antenatal care, pregnant people with complex social backgrounds or disabilities, or pregnant people with “chaotic lifestyles,” the definition of which ranged from those who did not regularly attend appointments to those with additional caring responsibilities at home. These data suggest a tension between pregnant people and healthcare professionals about the nature of those adjudications. While healthcare professionals would like to empower pregnant persons through participatory surveillance, skepticism remained about *capability*, a skepticism not borne out by the results of the UDIP study showing broad equivalence of ability between pregnant people and healthcare professionals (Jakubowski, Stevens, et al., 2022). However, some pregnant people were also uncertain about the loss of the traditional clinical gaze and preferred a level of clinical oversight to remain. While they did not necessarily think they were *incapable* of self-testing, they were reluctant for healthcare professionals to wholly relinquish the responsibility of surveillance activities. The conceptualization of the clinical gaze is complex in these data; pregnant people still wanted a “safety net,” for an element of the clinical gaze to remain even as they desired more control and agency in their antenatal care. Foucault’s conceptualization of surveillance goes one way—*them upon us*. Our findings suggest that self-testing does not necessarily remove this by handing power to pregnant people but amplifies the gaze, as self-testing is adding another layer of clinical surveillance. However, this further layer of surveillance is more participatory and empowering than traditional elements of antenatal care. Healthcare professionals and pregnant people expressed that self-testing was acceptable *because* it created additional clinical surveillance and simultaneously allowed pregnant people more control and agency in their antenatal care.

Both healthcare professionals and pregnant people considered self-testing empowering. The link between self-testing and patient empowerment has been explored in other areas of healthcare research, predominately the HIV testing space (Kapeller & Loosman, 2023; Ngure et al., 2017; Wachinger et al., 2021). To date, there has been relatively little research on empowerment and self-testing in maternal health; a recent systematic review by this team found relatively little data on self-management in pregnancy, outside of diabetes management (Jakubowski, Hinton, et al., 2022). A recent study on group antenatal care reflected on the inclusion of self-testing in these settings and how it enhanced participants’ feelings of control and safety (Hunter et al., 2019), but this was only a small component of the intervention, and more research is needed to explore self-testing/management and how it could relate to empowerment in antenatal care. Access to care is particularly important for women from minoritized or socially deprived groups as they have been repeatedly shown to have poorer maternity outcomes (Cresswell et al., 2013; Knight et al., 2022; Lindquist et al., 2015). A systematic review exploring the experiences in maternity care of pregnant women with social risk factors from Rayment-Jones and colleagues found that the candidacy of pregnant people was impacted by healthcare professionals’ assumptions based on race, socio-economic status, age, ability, and other “sources of oppression” (Rayment-Jones et al., 2019); these results are supported by the findings of this study. Rayment-Jones’ review also explored how paternalism impacts the candidacy of pregnant people with social risk factors. Our data show that medicalization, due to high-risk pregnancy, resulted in a tension between healthcare professionals and pregnant people regarding the negotiation of candidacy and the responsibility for surveillance.

The culture of risk avoidance in maternity care (Scamell, 2014) is also necessary to consider in light of the findings of this study. The perception of risk in pregnancy is shaped by the threat of litigation and the additional complexity of caring for a dyad, both the parent and the baby. Healthcare professionals, according to Scamell and Alaszewski, see continual surveillance as a necessary function of antenatal care because it could, at any moment, uncover a previously unknown risk to the pregnancy. A culture of risk avoidance lends itself to a more medicalized model of care. In maternity care, patient safety concerns are intertwined with litigation concerns; maternity claims dominate the cost of clinical negligence in the United Kingdom, contributing the most to clinical negligence costs, yet accounting for only 11% of the total number of claims received (NHS Resolution, 2019/20). Considering the ubiquitous system of surveillance and intervention involved with childbirth in settings such as the United Kingdom (Coxon et al., 2012), litigation consequences are steep when there is a poor outcome (Draycott et al., 2015). The views of healthcare professionals, and their hesitancies regarding self-testing, may also be colored by the overall impression that self-testing, using dipsticks, is not an accurate measure of assessing proteinuria in pregnancy when compared to automated dipstick readers (Correa et al., 2017; Waugh et al., 2005). However, the diagnostic accuracy study that predicated this study contrasts with this research and suggests dipstick testing is more accurate than previously thought (Jakubowski, Stevens, et al., 2022).

### Strengths and Limitations

Qualitative data on self-testing for proteinuria during pregnancy is sparse, and this study addresses this current gap in the literature. The strength of this study lies in the mixed-methods data collection, which adds both depth and breadth. Supplementing the healthcare professional interviews with focus groups allowed an exploration of topics that had been brought up in the interviews and allowed healthcare professionals to tease out thoughts and viewpoints from each other that the moderator, or interviewer, may not have been able to as an outsider to the profession and the group.

Some pregnant participants were inpatients, with the stress and vulnerability that entails. Interviews were often short and were sometimes ended prematurely due to clinical commitments. Inpatient interviews took place shortly after participants had completed the practical aspects of the UDIP study (i.e., testing their own urine) but were sometimes interrupted by healthcare professionals or partners and family of participants. Due to the busy and hectic nature of healthcare professionals’ work lives, interviews with them were often short and conducted in empty rooms on busy wards.

The group of pregnant people interviewed for this study were more likely to be positive about self-testing as they were willing to take part in the UDIP study, and thus not all discordant views on self-testing may have been captured. The authors are cognizant that those who are typically most underrepresented in maternity care research were not wholly captured in this study (Hinton et al., 2021), and that their views on self-testing and their experiences of antenatal care may differ from those represented in this study.

The data in this study were collected prior to the COVID-19 pandemic, and although it remained omnipresent in the write-up, the views and opinions on self-testing captured in this study do not fully reflect the changes to maternity care prompted by the pandemic. Face-to-face care has since resumed, yet the acceptability of remote care may have changed considering people’s experiences during the height of the pandemic where many antenatal appointments were conducted remotely (Jardine et al., 2021).

## Conclusions

Overall, pregnant people and healthcare professionals found self-testing, and with it the concept of participatory surveillance, acceptable. The equivalence of efficacy found in the diagnostic accuracy study emphasizes the fact that self-testing can be accurate and has the potential to be an empowering and beneficial intervention in antenatal care. However, these data illuminate tensions negotiating individual candidacy for participation in such interventions. Self-testing represents a shift in how antenatal care is delivered, as responsibility for surveillance is shared between healthcare professionals and pregnant people. The data reported in this paper reveal a tension between the desire to involve pregnant people more in their antenatal care via self-testing and the hesitancy, among both pregnant people and healthcare professionals, to lose the clinical gaze that has traditionally defined the practice of antenatal care surveillance. Further research is needed to understand how self-testing could be embedded into usual care, how shared responsibility for antenatal surveillance affects the relationship between healthcare professionals and pregnant people, and how to reduce the barriers pregnant people face accessing these types of interventions.

## Supplemental Material

Supplemental Material - Participatory Surveillance and Candidacy: A Discourse Analysis of Views on Self-Testing for Proteinuria in Pregnancy Supplemental Material for Participatory Surveillance and Candidacy: A Discourse Analysis of Views on Self-Testing for Proteinuria in Pregnancy by B. E. Jakubowski, K. L. Tucker, L. Lavallee, H. Wilson, L. Mackillop, L. C. Chappell, R. J. McManus, and L. Hinton in Qualitative Health Research
