# Supplementary material for: Participatory Surveillance and Candidacy: A Discourse Analysis of Views on Self-Testing for Proteinuria in Pregnancy
Source: Qual Health Res. 2024 Oct 17;35(8):863–75. doi: 10.1177/10497323241274270 (PMC12117135; doi:10.1177/10497323241274270)
Supplement: Supplemental Material - Participatory Surveillance and Candidacy: A Discourse Analysis of Views on Self-Testing for Proteinuria in Pregnancy [file sj-pdf-1-qhr-10.1177_10497323241274270.pdf]

Topic Guide: Healthcare Professionals

Hi, I'd like to talk to you today about urine testing in pregnancy, specifically about pregnant women self-testing their own urine. But I'd also like to know a bit about you, and I thought we could start off by chatting about your career and how long you've been in the profession?

1. What are your thoughts on the (name) study
2. Have you come across self-testing before the (name) study?
    - If so, ask to elaborate
3. What do you think of a home-based self-testing regime?
4. Do you think women testing at home will change your relationship with your patients?
5. Do you see any barriers to women self-testing?
6. Who do you think will benefit most from self-testing?

Topic Guide: Pregnant Women

1. In your own words, could you tell me a bit about your pregnancy journey so far?
2. Did you know anything about proteinuria before the (name) study?
    - Where did you find this out from?
3. What did you think of the (name) study?
4. Did you have any problems with the study?
5. What are your thoughts on self-testing your urine at home?
6. What do you think the benefits of self-testing would be for you?
7. Do you think there would be any barriers to you self-testing at home?
8. Do you think self-testing would change anything about your relationship with your clinician?
